# Sleep-State Dependent Alterations in Brain Functional Connectivity under Urethane Anesthesia in a Rat Model of Early-Stage Parkinson’s Disease

**DOI:** 10.1523/ENEURO.0456-18.2019

**Published:** 2019-02-26

**Authors:** Ekaterina Zhurakovskaya, Juuso Leikas, Tiina Pirttimäki, Francesc Casas Mon, Mikko Gynther, Rubin Aliev, Tomi Rantamäki, Heikki Tanila, Markus M. Forsberg, Olli Gröhn, Jaakko Paasonen, Aaro J. Jalkanen

**Affiliations:** 1 A. I. Virtanen Institute for Molecular Sciences, University of Eastern Finland, Kuopio, FI-70211, Finland; 2 School of Pharmacy, University of Eastern Finland, Kuopio, FI-70211, Finland; 3 Moscow Institute of Physics and Technology, 117303, Moscow, Russia; 4 Institute of Theoretical and Experimental Biophysics, 142292, Puschino, Russia; 5 Laboratory of Neurotherapeutics, Division of Pharmacology and Pharmacotherapeutics, Faculty of Pharmacy, University of Helsinki, Helsinki, FI-00790, Finland

**Keywords:** 6-OHDA lesion, connectivity, rat, resting-state fMRI, sleep, urethane

## Abstract

Parkinson’s disease (PD) is characterized by the gradual degeneration of dopaminergic neurons in the substantia nigra, leading to striatal dopamine depletion. A partial unilateral striatal 6-hydroxydopamine (6-OHDA) lesion causes 40–60% dopamine depletion in the lesioned rat striatum, modeling the early stage of PD. In this study, we explored the connectivity between the brain regions in partially 6-OHDA lesioned male Wistar rats under urethane anesthesia using functional magnetic resonance imaging (fMRI) at 5 weeks after the 6-OHDA infusion. Under urethane anesthesia, the brain fluctuates between the two states, resembling rapid eye movement (REM) and non-REM sleep states. We observed clear urethane-induced sleep-like states in 8/19 lesioned animals and 8/18 control animals. 6-OHDA lesioned animals exhibited significantly lower functional connectivity between the brain regions. However, we observed these differences only during the REM-like sleep state, suggesting the involvement of the nigrostriatal dopaminergic pathway in REM sleep regulation. Corticocortical and corticostriatal connections were decreased in both hemispheres, reflecting the global effect of the lesion. Overall, this study describes a promising model to study PD-related sleep disorders in rats using fMRI.

## Significance Statement

Disturbances in sleep patterns and rapid eye movement (REM) sleep behavior disorder are among the first symptoms of Parkinson’s disease (PD). However, PD-related sleep disorders have been virtually unexplored in animal models. This is a first study which has examined the functional connectivity (FC) during urethane-induced sleep-like states in the partial striatal 6-OHDA lesion rat model of early-stage PD using functional magnetic resonance imaging (fMRI). We found that FC is significantly decreased in 6-OHDA lesioned animals, but only during the REM-like state. These changes affected both the lesioned and intact hemispheres, mostly involving corticocortical and corticostriatal connections. The results suggest that this rat model is a promising tool for studying sleep disturbances in early-stage PD with resting-state fMRI.

## Introduction

Parkinson’s disease (PD) is the second most common neurodegenerative disease and the most common movement disorder affecting 1% of the population worldwide after the age of 70 years (Pringsheim et al., 2014). A gradual degeneration and loss of dopaminergic neurons in the midbrain substantia nigra pars compacta are the pathologic hallmarks leading to striatal dopamine (DA) depletion, resulting in progressive motor symptoms including bradykinesia, rigidity, and resting tremor, which are characteristic of PD (Schapira, 2009). In addition to the motor symptoms, PD patients suffer from a variety of non-motor symptoms, including sleep disorders, olfactory dysfunction, pain, depression, anxiety, impulsive behavior, and cognitive impairment, which significantly impact negatively on the patient’s quality of life (Titova and Chaudhuri, 2017).

PD patients may experience a variety of sleep disorders, such as restless legs syndrome, rapid eye movement (REM) sleep behavior disorder (RBD), sleep fragmentation, and insomnia (French and Muthusamy, 2016). Because sleep disorders appear several years before the appearance of the motor deficits (Bargiotas et al., 2016), they have been considered as an early marker for PD. In particular, the presence of RBD represents a considerably elevated risk of developing PD (Postuma et al., 2013). PD patients with RBD have increased α-synuclein deposition (Postuma et al., 2015), more severe initial motor symptoms, and they require higher levodopa doses as the disease progresses (Chung et al., 2017). It is notable that alterations in brain network organizations have been associated with RBD even before the onset of obvious motor impairment in PD (Ellmore et al., 2013). Thus, an experimental model mimicking early sleep disorders is needed in assessing potential treatments for these disorders and it may also represent a new approach for testing novel disease-modifying treatments for early-stage PD.

Unilateral lesioning of the substantia nigra dopaminergic neurons using the 6-hydroxydopamine (6-OHDA) is one of the most widely used animal models of PD (Bové and Perier, 2012). This lesion induces a gradual degeneration of nigrostriatal DA neurons and motor impairments resembling PD phenotypes. Depending on the administration protocol and infusion site of the toxin, a 6-OHDA lesion can produce very different degree of biochemical and behavioral outcomes. A near complete loss of striatal DA can be induced by infusing the toxin into the medial forebrain bundle (MFB) or directly into the substantia nigra (Lee et al., 1996; Yuan et al., 2005). This procedure leads to prominent neurodegeneration and severe motor deficits. Instead, an intrastriatal infusion of 6-OHDA produces a partial striatal DA depletion (usually a 20–80% DA deficiency depending on the 6-OHDA dose and number of infusion sites) accompanied by mild but progressive well characterized motor deficits (Sauer and Oertel, 1994; Przedbroski et al., 1995; Leikas et al., 2017). Thus, this partial 6-OHDA lesion is considered more relevant as a model for early-stage PD. Despite the relevance of sleep disturbances in early PD, the effects of the partial striatal 6-OHDA lesion on brain connectivity and sleep have not been well characterized. For example, it is not known whether the 6-OHDA lesioned rats express disturbances in brain activity during sleep resembling those encountered in PD patients.

Resting-state functional magnetic resonance imaging (rsfMRI) is a sophisticated MRI technique assessing functional connectivity (FC). It is typically based on the detection of correlating spontaneous low-frequency (<0.1 Hz) fluctuations in blood oxygenation level-dependent (BOLD) signals (Biswal et al., 1995); similar BOLD signal fluctuation profiles in different brain regions reflect synchronized neuronal activity and FC. It has proven its clinical value as a non-invasive imaging method for assessing disruptions in functional brain networks in patients with early and advanced PD (Nandhagopal et al., 2008; Lopes et al., 2016; Tahmasian et al., 2017).

Here, we used a unilateral partial striatal 6-OHDA lesion protocol producing ∼50% striatal DA depletion and investigated the functional brain network alterations in this rat model of early-stage PD by subjecting the animals to rsfMRI at 5 weeks after the 6-OHDA infusion. In particular, we focused on analyzing different urethane-induced sleep-like states (Clement et al., 2008; Wilson and Yan, 2010; Pagliardini et al., 2013) and based on the RBD symptomatology, we hypothesized that the potential brain connectivity changes would become manifest during the REM state of sleep. Furthermore, we measured the striatal levels of DA, GABA, and glutamate, the key neurotransmitters controlling the basal ganglia-thalamocortical motor network impaired in PD, to detect biochemical associations with the functional brain network alterations.

## Materials and Methods

### Animals

The study was conducted in twenty 6-OHDA lesioned, 10 sham-operated, and 10 naïve male Wistar rats supplied by the Laboratory Animal Center of the University of Eastern Finland (Kuopio, Finland). The rats were 8 weeks old and weighed 270–360 g at the beginning of the experiments. Unless otherwise stated, the rats were pair-housed in stainless steel cages and kept on a 12 h light/dark cycle (lights on at 07:00 A.M.) at an ambient temperature of 22 ± 1°C. Pelleted food (Teklad 2016S, Harlan Laboratories) and tap water were available *ad libitum*. All procedures were made in compliance with ARRIVE guidelines and the European Commission Directive 2010/63/EU and were approved by the Finnish National Animal Experiment Board (licenses ESAVI-2014-701, ESAVI-2013-00833). All effort was taken to minimize the number of animals used and their suffering.

### Drugs

6-OHDA·HCl (Sigma-Aldrich) was dissolved in vehicle (0.9% NaCl containing 0.2 mg/ml ascorbic acid). Pentobarbital (Mebunat vet, Orion Oyj) and buprenorphine (Schering-Plow) were dissolved in saline, and urethane (Sigma-Aldrich) in sterile water. Lidocaine (Xylocaine) was obtained from AstraZeneca. The doses of drugs refer to the free bases.

### Partial unilateral 6-OHDA lesion model of early-stage PD

The rats were anesthetized with pentobarbital (55 mg/kg, i.p.). Lidocaine was applied to the scalp and to the surface of the skull and a single dose of buprenorphine (0.02 mg/kg, s.c.) was given to relieve any postoperative pain. Then, 6-OHDA (10 µg/4 µl, *n* = 20) or vehicle (i.e., sham lesion, *n* = 10) was infused into the right striatum (coordinates from bregma: AP: +1.0, L: −2.7, DV: −5.0; Paxinos and Watson, 2007) at a flow rate of 0.5 µl/min. The needle was retained in position for 4 min after the infusion to prevent backflow. After the surgery, the animals were kept in individual stainless steel cages for 3 d to allow recovery and subsequently returned in groups of two. The success of the lesion was verified by striatal DA analysis after rsfMRI imaging. Two animals were excluded from the study based on an abnormal striatal DA ratio: (1) no DA depletion was observed in lesioned striatum indicating a failed 6-OHDA administration (lesion/intact DA ratio >13× SEM vs group mean), and (2) an abnormal decrease in striatal DA ratio was observed in one sham lesioned rat (lesion/intact DA ratio <6× SEM vs group mean). One 6-OHDA lesioned animal was excluded from the study because of an abnormal finding in the anatomic MRI image (possible cyst or effusion). Furthermore, one sham lesioned animal was excluded from the biochemical data analysis because of an error occurring during tissue sample preparation.

### fMRI

fMRI was done with 7T Bruker Pharmascan MRI system (Bruker Biospin) 5 weeks after 6-OHDA or sham lesion when the rats were 13 weeks old. Naïve animals were imaged in the beginning of the experiments at 8 weeks of age. A rat brain surface and a resonator volume coil were used for signal reception and transmission, respectively (both quadrature coils from Bruker Biospin). Rats were anesthetized with urethane (1250–1500 mg/kg, i.p.), and subsequently fixed to an MRI rat holder with ear bars and a bite bar. The rsfMRI data were acquired with single-shot spin-echo echoplanar imaging sequence with the following parameters: repetition time: 2000 ms, echo time: 45 ms, 1200 volumes (40 min), field-of-view: 32 × 32 mm, 17 slices with a thickness of 0.9 mm, matrix size 64 × 64, and bandwidth 200 kHz.

An MRI-compatible small animal monitoring system (Model 1025, Small Animal Instruments), including rectal temperature probe, respiration pneumatic sensor, and fiber-optic oximetry sensor, was used to monitor animal physiology. The rats were kept warm (∼37°C) by a built-in water circulation system (ThermoFisher Scientific).

**Figure 1:**
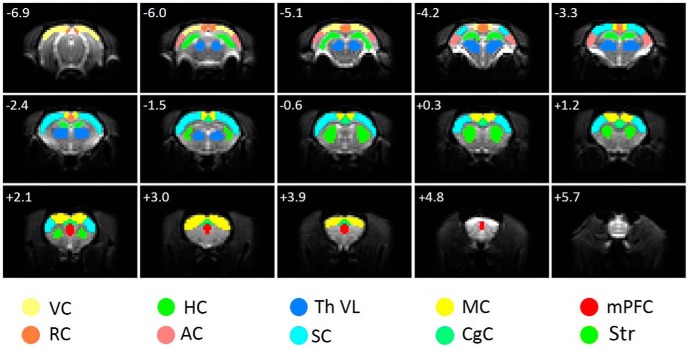
ROIs used for FC analysis. ROIs are overlaid on original spin-echo echoplanar imaging functional MRI images. AC, Auditory cortex; CgC, cingulate cortex; HC, hippocampus; MC, motor cortex; mPFC, medial prefrontal cortex; RC, retrosplenial cortex; SC, somatosensory cortex; Str, striatum; Th VL, ventral lateral thalamus; VC, visual cortex. Distance to bregma in millimeters is marked in the top left corner of each slice.

Before the analyses, the rsfMRI data were converted to NIfTI using in house written MATLAB code (http://aedes.uef.fi), slice-timing corrected, motion-corrected, spatially smoothed (2 × 2 voxel full-width at half-maximum Gaussian kernel), and coregistered by using SPM8 (www.fil.ion.ucl.ac.uk/spm) and MATLAB v2017b (MathWorks). The FC analyses were done with an in-house MATLAB code, Aedes, and SPM8. Seventeen regions-of-interest (ROIs) were drawn according to an anatomic atlas (Paxinos and Watson, 2007; Fig. 1). Subsequently, the rsfMRI data were bandpass filtered at 0.01–0.08 Hz to minimize physiologic and hardware-induced noise.

To obtain measures for FC, partial correlation coefficients between ROIs (or ROI and voxels in correlation maps) were calculated by using MATLAB. All six motion correction parameters, obtained with SPM, were used as regressors in the correlation calculations to minimize the effect of motion on the results.

### Assessment of sleep states and FC

Fluctuations in breathing rate during fMRI were used as indirect measures to detect spontaneous sleep-like states as described by (Wilson et al., 2011; Pagliardini et al., 2012). States with high and low breathing rates were manually labeled as REM-like and non-REM (NREM)-like sleep states, respectively. Animals with transitions between NREM-like and REM-like state were included in the sleep-state analysis. The state-labeled fMRI data were divided into windows of size of 30 volumes. State-labeled partial correlation coefficients were calculated for each window, and subsequently averaged within each subject. If an animal did not have at least 90 fMRI volumes of sleep-like state in total, it was excluded from the sleep-state analysis.

### Dissection of brain samples and biochemical analyses

6-OHDA and sham lesioned animals were killed by decapitation under urethane anesthesia immediately after the rsfMRI measurements. After decapitation, the brains were rapidly removed and both striata were dissected on ice, frozen, and stored at −80°C until analyzed. The striatal samples were homogenized (Soniprep 150 MSE Scientific Instruments) in 25 volumes of ice-cold 0.1 m perchloric acid (Merck KGaA). The homogenates were centrifuged at 15000 × *g* (4°C, 15 min, Heraeus Sepatech, Biofuge 17RS). The supernatant was collected and filtered (GHP Acrodisc 13, 0.45 µm) into Eppendorf tubes. Supernatants were further diluted twofold with 0.1 m perchloric acid before DA analysis and 350-fold with acetonitrile (CAN; Mallinckrodt Baker) before GABA and glutamate analysis. DA levels in both striata were analyzed by a high performance liquid chromatography method with electrochemical detection (Kääriäinen et al., 2008). Striatal GABA and glutamate levels were measured with a liquid chromatography/mass spectrometry (LC-MS/MS) method. Liquid chromatography was performed on Agilent 1290 Infinity LC (Agilent Technologies) instrumentation. GABA and glutamate were separated using Waters Acquity UPLC BEH amide column (100 × 2.1 mm, 1.7 µm). The column temperature was maintained at 40°C. The A mobile phase was 40 mm ammonium formate buffer + ACN (1:1) and the B mobile phase was 200 mm ammonium formate buffer + ACN (1:10). A gradient was applied for over 12 min as follows: from 0 to 6 min 95–70% B, from 6 to 7 min, 70–95% B, and finally the column was equilibrated from 7 to 12 min at 95%. The flow rate was 0.3 ml/min and the injection volume was 5 μl. The LC-MS/MS data were acquired with an Agilent 6410 Triple Quadrupole Mass Spectrometer (Agilent Technologies) using Multiple Reaction Monitoring applying the following conditions: electrospray ionization, positive ion mode; drying gas (nitrogen) temperature, 200°C; drying gas flow rate, 16 L/min; nebulizer pressure, 25 psi; and capillary voltage, 4500 V. Quantitative transitions were 104 → 87 and 148 → 130 (m/z) for GABA and glutamate, respectively. The fragmentor energy was 380 V and collision energy was 7 V for both GABA and glutamate. Biochemical analyses were not performed on the naïve animals.

Striatal DA, GABA, and glutamate ratios were calculated with the equation *C*_lesioned side_/*C*_intact side_.

### Statistical analysis

All results are expressed as mean ± SD. Differences in DA, GABA, and glutamate levels in lesioned versus intact striatum were tested with two-tailed paired *t* test using GraphPad Prism 5.03 software. The physiologic parameters were compared between groups using one-way ANOVA with Tukey’s multiple-comparison test with GraphPad Prism software. The comparison of sleep-like states durations was done using two-tailed unpaired *t* test with in-house MATLAB codes. The correlation coefficients were normalized with Fisher’s *z*-transformation. The group comparison for ROI-based and seed-based analysis were performed using two-tailed unpaired *t* test with false discovery rate (FDR) correction for multiple comparisons with in-house MATLAB codes. The criterion for statistical significance was set at *p* < 0.05 for all tests.

## Results

### The effect of the partial striatal 6-OHDA lesion on striatal levels of dopamine, GABA, and glutamate

**Figure 2:**
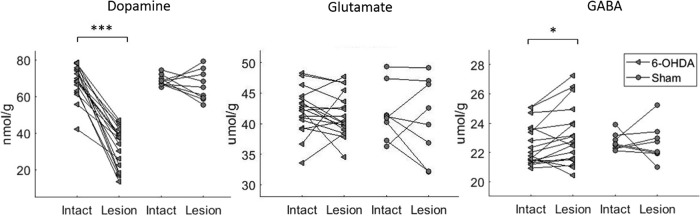
Dopamine (nmol/g tissue), glutamate, and GABA levels (µmol/g tissue) in the intact and lesioned striatum in 6-OHDA lesioned and sham animals. Paired *t* test: *p < 0.05, ***p < 0.001.

The effects of 6-OHDA and sham lesions on striatal levels of biochemical markers are shown in Figure 2. As expected, the partial striatal 6-OHDA lesion (*n* = 18) depleted striatal DA levels by 51% in the lesioned side (33.2 ± 10.5 vs 67.9 ± 9.0 nmol/g of tissue weight in lesioned and intact striatum, respectively; paired *t* test, *p* < 0.0001). Additionally, the 6-OHDA lesion induced a slight but significant increase in striatal GABA levels (23.1 ± 2.0 vs 22.5 ± 1.4 µmol/g of tissue weight in lesioned and intact striatum, respectively; paired *t* test, *p* < 0.05). In contrast, the 6-OHDA lesion had no effect on striatal glutamate levels (41.4 ± 3.4 vs 42.2 ± 3.5 µmol/g of tissue weight in lesioned and intact striatum, respectively). The sham lesion (*n* = 8) exerted no significant effect on striatal DA (64.4 ± 7.1 and 69.3 ± 2.8 nmol/g of tissue weight in sham-lesioned and intact striatum, respectively, n.s.), GABA (22.7 ± 1.3 vs 22.7 ± 0.6 µmol/g of tissue weight in sham-lesioned and intact striatum, respectively, n.s.) or glutamate (40.8 ± 6.2 vs 41.7 ± 4.2 µmol/g of tissue weight in lesioned and intact striatum, respectively, n.s.) levels.

### Animal physiology during fMRI measurements

There were no statistical differences between the 6-OHDA and sham lesioned animals in any of the physiologic measures [breathing rate (125 ± 21 vs 128 ± 19, breaths/min, sham vs 6-OHDA, respectively), heart rate (358 ± 32 vs 380 ± 37 beats/min), core body temperature (37.1 ± 0.4 vs 37.0 ± 0.5°C), and weight (418 ± 25 vs 412 ± 29 × *g*)] during the onset of the fMRI measurement. In the naïve group, the corresponding physiologic parameters were as follows: breathing rate 107 ± 12 breaths/min, heart rate 349 ± 39, temperature 36.7 ± 0.6°C, and weight 316 ± 22 g, i.e. naïve animals had lower weights and also a lower breathing rate than those of lesioned and sham animals (both parameters *p* < 0.05, one-way ANOVA with Tukey’s test), but all parameters are within the normal range.

### The effects of the unilateral partial striatal 6-OHDA lesion on overall connectivity

**Figure 3:**
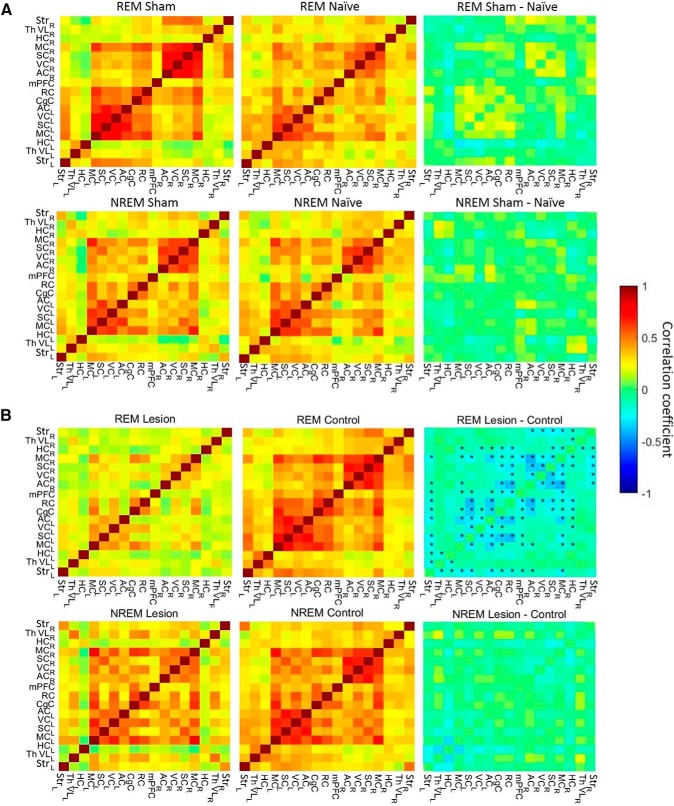
FC between the brain regions in naïve and sham animals (***A***). There were no statistically significant differences after FDR correction for multiple comparisons between sham and naïve groups. FC between the brain regions in 6-OHDA and control animals (***B***). Top, Connectivity matrices for the REM-like state. Bottom, Connectivity matrices for the NREM-like state. Left to right, Mean connectivity matrix in 6-OHDA lesioned animals, mean connectivity matrix in control animals, the difference between the first two panels. A higher absolute correlation coefficient corresponds to higher connectivity between regions. In the difference pictures (top right), stars denote statistically significant differences between groups (*p* < 0.05, FDR-corrected for multiple comparisons). Abbreviations for brain regions as in Figure 1.

First, we investigated whether the sham lesion would affect the FC by comparing the region–region connectivity of the sham group to that of the naïve animals. Although there were some weight and breathing differences between the sham and naïve animals, there were no differences in the resting-state FC (all *p* values > 0.2, unpaired *t* test, FDR-corrected; Fig. 3*A*), evidence of the robustness of the urethane-induced REM/NREM-like FC states. Because the sham lesion had no effect on the striatal biochemical markers nor the resting-state FC, we combined the naïve and sham groups and refer to it as the control group (*n* = 18) from here onward.

Next, we compared the resting-state FC obtained from the whole fMRI measurement period between the 6-OHDA lesioned and control groups. This analysis revealed no significant differences in FC between the 6-OHDA lesioned and control animals in overall connectivity; only trends toward increased FC from the right to the left ventral lateral thalamus (*p* = 0.048, not FDR-corrected) and from the right ventral lateral thalamus to the cingulate cortex (*p* = 0.040, not FDR-corrected) were observed. In addition, there was a trend toward decreased FC from ipsilateral to contralateral striatum (*p* = 0.047, not FDR-corrected). After correction for multiple comparisons, however, these trends become nonsignificant.

### FC during different sleep states

The fact that REM sleep is more active and energy-demanding state (Steriade and Hobson, 1976; Franzini, 1992; Lenzi et al., 2000) than NREM sleep and the proposition that REM-sleep associated sleep disorders are early symptoms of progressive neurodegeneration in PD patients (Heller et al., 2017) prompted us to separate REM and NREM sleep-like states in our rsfMRI analysis. Based on the breathing rate fluctuations (Wilson et al., 2011), we observed clear state changes between REM-like and NREM-like state in 8/19 control animals and 8/18 lesion animals. In the rest of the animals, only one sleep state was observed, and they were excluded from the sleep-state FC analysis. The lack of sleep-state transitions in approximately one-half of the animals may be explained by differences in the depth of anesthesia (Gretenkord et al., 2016) or factors influencing respiratory function, such as the environmental temperature (Whitten et al., 2009) or inhaled oxygen (Pagliardini et al., 2013; Hauer et al., 2018). The average durations for the REM-like and NREM-like states were 99 ± 80.4 and 78.6 ± 70.8 s, respectively. There were no significant differences in state durations between control and 6-OHDA lesioned animals (*p* = 0.96 for REM-like states, *p* = 0.84 for NREM-like states, unpaired *t* test).

**Figure 4:**
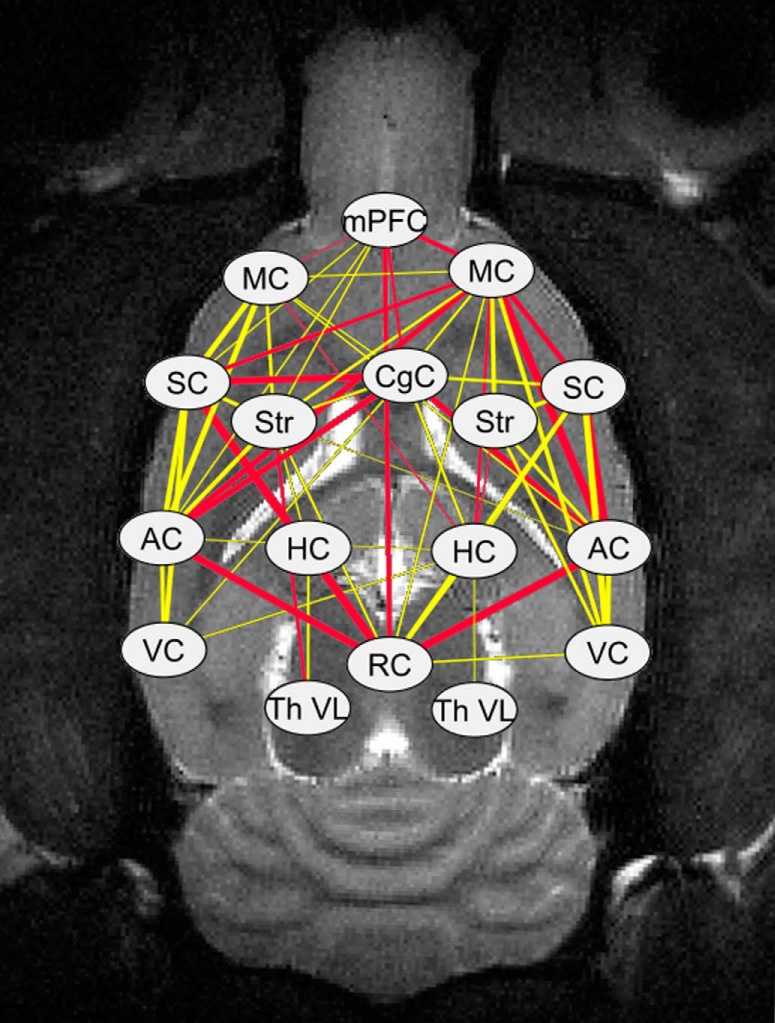
Significantly decreased (*p* < 0.05, FDR-corrected for multiple comparisons) connections between brain regions during REM-like state in rats with partial striatal 6-OHDA lesion. The width of the line corresponds to the mean correlation difference between control and lesioned groups: a thicker line represents a greater decrease in the correlation of the lesioned group. The differences in correlation coefficients range from 0.12 to 0.41. Yellow lines represent *p* values from 0.01 to 0.05; red lines, *p* values < 0.01. Abbreviations for brain regions as in Figure 1.

The FC differences between REM-like and NREM-like states in the control group had the same pattern as displayed by healthy rats in a previous study (Zhurakovskaya et al., 2016): increased thalamocortical connectivity during REM-like state and increased corticocortical connectivity between hemispheres during the NREM-like state. However, FC was disturbed in partially 6-OHDA lesioned animals compared with control rats. Interestingly, these disturbances were strongly dependent on the sleep-like state (Fig. 3*B*). During the REM-like state, corticocortical, striatocortical and corticohippocampal connections were substantially decreased in 6-OHDA lesioned animals (*p* < 0.05, FDR corrected; Figs. 3*B*, 4). In contrast, during the NREM-like state, there were no differences in the FC between the control and lesioned animals (all *p* values > 0.8).

**Figure 5:**
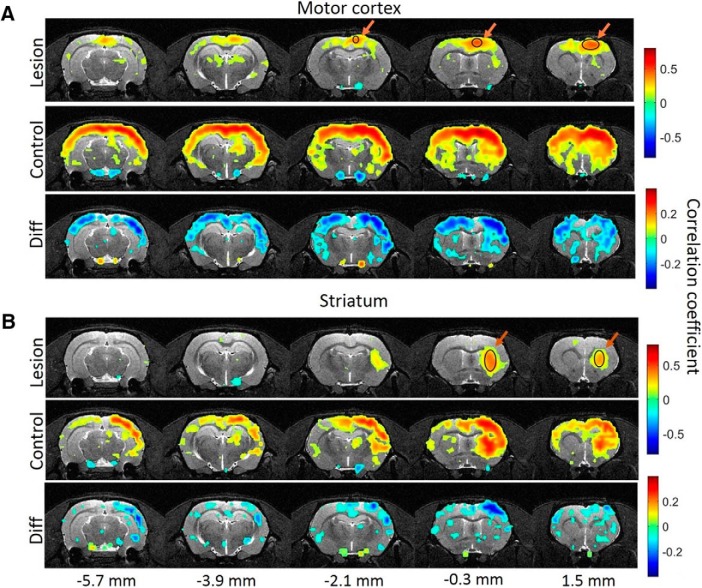
FC maps during REM-like state with seed region in right motor cortex (***A***) and right striatum (***B***). Top row, Group connectivity map for lesioned animals (statistically different from 0 correlations, *p* < 0.05, FDR-corrected for multiple comparisons); middle row, group connectivity map for control animals; bottom row, statistically significant differences (*p* < 0.05, FDR-corrected for multiple comparisons) between the first two. The seed region is marked with arrows and circles in the group map of lesioned animals. Distance to bregma for each slice is marked at the bottom.

The seed-based voxelwise analyses, obtained from ipsilateral motor cortex and striatum (Fig. 5), illustrate the 6-OHDA-induced extensive striatocortical and subsequent corticocortical disruptions in FC during the REM-like sleep state. No differences were observed during the NREM-like state (data not shown).

## Discussion

Intrastriatal infusion of 6-OHDA produces a gradual degeneration of the nigrostriatal dopaminergic pathway and a partial DA depletion in the striatum accompanied with behavioral manifestations resembling early-stage PD. The behavioral outcomes of the partial 6-OHDA model have been thoroughly characterized (Sauer and Oertel, 1994; Przedbroski et al., 1995; Leikas et al., 2017), and here, we show that the partial striatal DA depletion induces sleep-state-dependent changes in FC between several brain regions during urethane-induced sleep.

The observed DA deficiency (−51%) 5 weeks after infusion of a single 10 µg intrastriatal 6-OHDA dose is well in line with earlier studies using a similar 6-OHDA administration protocol (Lee et al., 1996; Leikas et al., 2017). Importantly, the extent of the striatal DA depletion in the present model is similar to that of the patients with an early clinically detectable PD (Schapira, 2009), highlighting the relevance of this model as an experimental model of early PD. In addition, 6-OHDA induced a small but significant increase in striatal GABA levels, which is in accordance with earlier studies in experimental models of PD (Tanaka et al., 1986; Chassain et al., 2008), as well as with studies in PD patients using *in vivo* MRI (Emir et al., 2012) or postmortem tissue level GABA analysis (Kish et al., 1986). In contrast, the 6-OHDA lesion had no effect on striatal GLU levels, which is consistent with an earlier *in vivo* study conducted in 6-OHDA lesioned rats (Tanaka et al., 1986), and parallels studies in PD patients (Taylor-Robinson et al., 1999; Kickler et al., 2007).

Our initial rsfMRI analysis on overall FC, obtained from the whole fMRI data, revealed only trends toward increased connectivity in some thalamocortical connections and between the ipsilateral and contralateral striatum. This result is in line with a very recent study in partially 6-OHDA lesioned rats showing a modest increase in FC in ipsi-lesioned and contra-lesioned regions of the corticobasal ganglia network at 3 weeks after the lesion (Perlbarg et al., 2018). The overall weaker changes in connectivity strengths observed in our study are most likely attributable to the smaller 6-OHDA dose (1 × 10 µg vs 2 × 10 µg) resulting in a lower degree of nigrostriatal dopaminergic damage in our model. In addition, a few recent studies have shown FC changes occurring in rats receiving higher 6-OHDA dose producing a complete loss of striatal DA. As expected, the high degree of dopaminergic damage induced by an MFB lesion has more prominent effects on FC, as indicated by reduced FC between the striata and increased FC between the lesioned striatum and cortical areas in both hemispheres (Monnot et al., 2017), and by increased cross-hemispheric thalamic FC and a general reduction in connectivity in the ipsilateral hemisphere and especially in cortical areas (Westphal et al., 2017). These clear differences between the partial and full lesion models are most likely attributable to the different degree of nigrostriatal dopaminergic damage. Furthermore, more prominent compensatory mechanisms in the MFB model may play a role in these phenomena. These mechanisms include increased bursting activity of the subthalamic nuclei (Janssen et al., 2012) and activation of alternative motor pathways (Eckert et al., 2006; Mallol et al., 2007) that attempt to compensate for the absence of ipsilateral nigrostriatal DA in the MFB model. Altogether, the present study further supports the less severe nature of the partial 6-OHDA lesion model and its relevance as a model for early-stage PD.

The sleep disturbances associated with early PD stimulated us to investigate whether the partial striatal 6-OHDA lesion rat model and urethane-induced sleep-like states could be a relevant approach for studying the pathophysiology of PD-related sleep disorders, particularly RBD, which have been considered as an early marker for PD because these sleep-related symptoms may appear even several years before the motor deficits (Postuma et al., 2013; Bargiotas et al., 2016). Sleep disorders are also associated with altered brain FC (Ellmore et al., 2013). Because natural sleep cannot be investigated with preclinical rsfMRI, recent studies have exploited different pharmacological tools to elicit sleep-like state (Wilson and Yan, 2010; Zhurakovskaya et al., 2016). Urethane anesthesia can be considered a practical model of natural sleep, because it is claimed to uniquely induce similar EEG and respiratory characteristics and sleep-like states as natural sleep in rodents. Additionally, urethane provides stable anesthesia for several hours with a normal physiologic state as reflected in the normal values of blood gases (Paasonen et al., 2016), while only minimally modulating multiple neurotransmitter systems, several brain regions, and autonomic functions (Maggi and Meli, 1986; Hara and Harris, 2002), making it an ideal anesthetic agent to be exploited in rsfMRI studies (Paasonen et al., 2018). Furthermore, the connectivity differences between states in urethane-anesthetized rats resemble the changes in the connectivity patterns occurring in natural sleep in humans (Zhurakovskaya et al., 2016).

In the present study, the sleep-like state rsfMRI connectivity patterns were similar in control animals as previously described in healthy rats under urethane anesthesia (Zhurakovskaya et al., 2016), indicating that these animals can serve as a valid control for detecting changes in connectivity patterns. We found that brain FC patterns differed between striatal 6-OHDA-lesioned and control rats, but the differences between the groups were significant only during the REM-like sleep state. During the REM-like sleep, the 6-OHDA lesion caused extensive disturbances in FC in intracortical areas but also between the cortical areas and the striatum and hippocampus. Notably, reductions in FC occurred in both hemispheres suggesting that the unilateral striatal DA depletion significantly also disturbed interhemispheric connections during the REM-like sleep. The disruptions in striatocortical signaling are not surprising considering the key role of striatal DA in controlling the cortico-striato-thalamo-cortical motor circuitry; according to this classical basal ganglia circuitry model of PD, DA controls the release of GABA in striatum, and thus regulates the activity of the basal ganglia output nuclei. In PD, the striatal DA depletion causes increased GABAergic inhibitory control in thalamus leading to over-inhibition of the thalamocortical glutamatergic signaling, and finally, to motor dysfunction (Centonze et al., 1999; Blandini et al., 2000; DeLong and Wichmann, 2007). In support for the dopaminergic control of GABA release in striatum, we also observed a slight increase in striatal GABA levels in 6-OHDA lesioned rats. The over-inhibition of the cortico-striato-thalamo-cortical circuitry could be reflected as reduced FC between the striatum and cortical areas. One could also speculate that the observed intracortical disruptions are a downstream consequence of the impaired striatocortical signaling.

It is noteworthy that we did not observe any significant 6-OHDA lesion-dependent changes in FC without differentiating between the different sleep-like states. Thus, it appears that the categorization of data based on sleep-like states improves the sensitivity of the rsfMRI analysis. The presence of the significantly impaired striatocortical connectivity only during the REM-like state could be explained by an overall decrease in cortical effective connectivity during the NREM-like state, which is partially restored during the REM-like state (Massimini et al., 2010). In other words, the silencing of long-range connectivity from cortex during the NREM state might hinder the observation of striatocortical connectivity changes. However, this explanation does not apply to the changes in corticocortical connectivity. Another possibility for the altered FC during different states of sleep is the direct participation of DA in the regulation of sleep. Striatal 6-OHDA injections induce retrograde dopaminergic cell death in substantia nigra pars compacta (Penttinen et al., 2016), and it is well established that a loss of these neurons disturbs the sleep patterns of rats (Lima et al., 2007), including changes in the regulation of muscle tone during REM sleep (Vo et al., 2014). This may be reflected in alterations in the connectivity between motor and somatosensory cortex and other cortical areas, as well as striatocortical connectivity during urethane-induced sleep.

It is interesting to note that the 6-OHDA-induced changes in FC were not restricted to the classical PD-associated cortico-striato-thalamo-cortical circuitry during the REM-like sleep. In particular, ipsilateral and contralateral hippocampal connections with the striatum, the cingulate cortex, and the ventral lateral thalamus were impaired by the striatal DA depletion. The disturbances in hippocampal connections may be associated with the well established memory impairing effects of the 6-OHDA lesion (More et al., 2016). It is unclear how striatal DA loss regulates hippocampal connections because there is no direct pathway connecting the dorsal striatum and hippocampus. However, FC may occur between any brain structures that are connected by large distributed networks. For instance, thalamus plays a central role in the complex network connecting the basal ganglia and cortical areas (Lanciego et al., 2012), and the hippocampus receives inputs from the thalamus. These excitatory inputs may well be affected by the increased GABAergic inhibitory control from the basal ganglia and explain the decreased hippocampal connectivity in 6-OHDA rats. Furthermore, the hippocampus has direct connections to the association cortex and to the entorhinal cortex, which has reciprocal projections to the cingulate cortex (Kerr et al., 2007). The cingulate cortex is further connected to wide cortical areas, and through this pathway, the hippocampus is also connected to many cortical areas. Therefore, it can be hypothesized that altered striatocortical connectivity could trigger disruptions in the balance of intracortical FC, which could be reflected as disturbances in corticohippocampal connectivity.

Non-invasive imaging and FC analysis are effective tools in the comparison of animal disease models and human pathology. In our study, the corticocortical and striatocortical connectivities were significantly decreased in 6-OHDA-lesioned animals during the REM-like sleep state. Decreased connectivity within the basal ganglia circuitry has been detected in patients with early stage PD (Szewczyk-Krolikowski et al., 2014; Dayan and Browner, 2017). Furthermore, the decreased FC from putamen to sensorimotor cortex and contralateral putamen has been observed already in an early stage of the disease (Luo et al., 2014). In patients with mid-stage and advanced stage of PD, decreased corticocortical and striatocortical connectivity has been reported in several studies (Hacker et al., 2012; Sharman et al., 2013). However, to confirm the translational value of the present results, the imaging should be conducted in conscious 6-OHDA lesioned animals. In addition, there is always variability in clinical studies in patients’ disease severities, pharmacotherapies, and ages, which introduces uncontrollable dispersion to data and complicates the comparison between animal studies and clinical findings. Despite the obvious differences between species and challenges in data acquisition in rodent rsfMRI compared with human studies, intrastriatally administered 6-OHDA, i.e., producing ∼50% striatal DA depletion, appears to induce the FC changes resembling those present in PD patients. Hence, these findings improve the construct validity of the partial striatal 6-OHDA lesion model.

Rodents with various nigrostriatal 6-OHDA lesions have been reported to display similar disturbances in sleep architecture as observed in PD patients. For example, rats with unilateral MFB lesion encounter increased muscle activity and limb movements during REM sleep (Vo et al., 2014) resembling those in PD patients. Furthermore, rats with bilateral partial striatal 6-OHDA lesion spend more time awake and have more frequent sleep-state transitions that induce limb movements and trigger arousals from sleep (Decker et al., 2000). In addition, bilaterally administered intrastriatal 6-OHDA disrupts endogenous circadian rhythm in mice (Masini et al., 2017). Altogether, these behavioral findings further support the applicability of nigrostriatal 6-OHDA lesion in modeling PD-related sleep disorders.

In conclusion, this study shows that the partial unilateral striatal 6-OHDA lesion altered brain FC patterns when rats were in a urethane-induced REM-like sleep state. Therefore, the present model can serve as a valid tool to investigate the REM-sleep associated disorders characteristic of early PD.

## Synthesis

Reviewing Editor: Julie Andersen, Buck Institute Decisions are customarily a result of the Reviewing Editor and the peer reviewers coming together and discussing their recommendations until a consensus is reached. When revisions are invited, a fact-based synthesis statement explaining their decision and outlining what is needed to prepare a revision will be listed below. The following reviewer(s) agreed to reveal their identity: Hossein Aleyasin, Kiyoshi Ando.

Both the reviewers and I concur that this manuscript focuses on an important unmet need in the Parkinson's disease (P{D) field--models for early non-motor changes in sleep disturbance prior to presentation of motor symptoms in the disorder. In this regard, the finding of a change in functional connectivity (FC) between 6-OHDA group and controls which becomes evident during the REM stage of urethane induced sleep-like state is certainly an exciting finding. In order to provide validity as a model of human disease, it would be important to provide evidence of REM disorder, for example, increased motor activity during REM-like state in the lesioned rats as has been shown to occur in PD patients. Although a lack of observable difference between biochemical markers and resting state FC is given as a reason for the pooling of sham-injected and naïve rats, we felt that these data should still be shown separately in order to validate. Questions also arose as to whether FC changes occur when dopaminergic neurons are highly lesioned by 6-OHDA or if induced by other anesthetics. If no,it is recommended that the title be altered to reflect this, for example “Alterations in overall (or global) brain functional connectivity in the urethane-anesthetized rat pretreated with unilateral infusion of 6-hydroxydopamine”. On a minor note, it is highly recommend to present biochemical analysis and neurotransmitter levels in a figure with multiple panels.

## Author Response

Comment: Both the reviewers and I concur that this manuscript focuses on an important unmet need in the Parkinson's disease (PD) field--models for early non-motor changes in sleep disturbance prior to presentation of motor symptoms in the disorder. In this regard, the finding of a change in functional connectivity (FC) between 6-OHDA group and controls which becomes evident during the REM stage of urethane induced sleep-like state is certainly an exciting finding.

Response: We thank the Reviewing Editor and the Reviewers for these encouraging comments.

Comment: In order to provide validity as a model of human disease, it would be important to provide evidence of REM disorder, for example, increased motor activity during REM-like state in the lesioned rats as has been shown to occur in PD patients.

Response: This is an important comment. Indeed, there is evidence that rodents with varying degree of 6-OHDA lesions encounter similar sleep-time motor disturbances as PD patients (e.g. increased muscle activity and limb movements during REM state and increased arousal from sleep). To address this comment, we have added a short discussion (page 16, rows 392-400) regarding these 6-OHDA associated changes in sleep architecture that resemble PD.

Comment: Although a lack of observable difference between biochemical markers and resting state FC is given as a reason for the pooling of sham-injected and naïve rats, we felt that these data should still be shown separately in order to validate.

Response: We have now compiled the new figure 3A to illustrate that there are no significant differences in resting state FC between the sham-lesioned and naïve rats as requested. Regarding the biochemical markers, data was unfortunately not obtained from naïve animals. However, dopamine/GABA/GLU levels are likely to be similar across the brain hemispheres in bilateral brain structures such as the striatum in healthy (intact) rats.

Comment: Questions also arose as to whether FC changes occur when dopaminergic neurons are highly lesioned by 6-OHDA or if induced by other anesthetics. If no, it is recommended that the title be altered to reflect this, for example “Alterations in overall (or global) brain functional connectivity in the urethane-anesthetized rat pretreated with unilateral infusion of 6-hydroxydopamine”.

Response: The effects of a higher degree of dopaminergic lesion by 6-OHDA on global functional connectivity have been investigated in papers by Perlparg (2018), Monnot (2017) and Westphal (2017) as discussed in the manuscript (3rd paragraph of the Discussion section). To emphasize the higher degree of dopaminergic damage in these previous papers, we have rephrased the text on rows 292-294.

The current study was performed in urethane-anesthetized rats, and there are several factors that support our choice:

1) Urethane is a specific anaesthetic for inducing the sleep-like states

2) Urethane provides a stable anaesthesia while only minimally modulating multiple neurotransmitter systems, several brain regions, and autonomic functions

3) Electrophysiological recordings show that urethane anesthesia has distinctively similar properties to natural sleep compared to several other anaesthetics

To justify the use of urethane in the present study, we have added a short discussion (and references) summarizing these points to the Discussion section (page 13, rows 312-318).

In addition, to better describe the experimental setup used, we have rephrased the title as “Sleep-state dependent alterations in brain functional connectivity under urethane anesthesia in a rat model of early-stage Parkinson's disease”.

Comment: On a minor note, it is highly recommend to present biochemical analysis and neurotransmitter levels in a figure with multiple panels.

Response: To elaborate the biochemical data, we have now compiled the new figure 2 to summarize the biochemical results, as suggested by the Reviewers.
